# Nonpharmacological interventions for the management of visuospatial disturbances in dementia in UK & Japan

**DOI:** 10.1002/alz70858_103943

**Published:** 2025-12-25

**Authors:** Maki Suzuki, Matthew J Bancroft, Sebastian J Crutch, Keir X X Yong, Manabu Ikeda

**Affiliations:** ^1^ Osaka University United Graduate School of Child Development, Suita, Osaka, Japan; ^2^ Centre for Vestibular and Behavioural Neurosciences, UCL Queen Square Institute of Neurology, London, United Kingdom; ^3^ Dementia Research Centre, UCL Queen Square Institute of Neurology, London, United Kingdom; ^4^ Osaka University Graduate School of Medicine, Suita, Osaka, Japan

## Abstract

**Background:**

Visuospatial disturbances caused by dementia severely affect autonomy by limiting activities like reading, object recognition, and mobility. These disturbances are prominent in posterior cortical atrophy (PCA), a neurodegenerative syndrome characterized by significant atrophy in the posterior brain regions. In this presentation, we will present technology‐based evidence for managing dementia‐related visuospatial disturbances, contextualized by examples of multidisciplinary support from UK and Japanese centers.

**Method:**

We focused on two core visuospatial features: space perception difficulties and environmental agnosia. UCL participants underwent laboratory‐based tasks involving object localization and mobility, assessed using kinematic measures. They were repeatedly asked to locate objects surrounded by distractors varying in contrast, shape and distance relative to the target (PCA *n* = 21; typical AD *n* = 17; Control *n* = 18) and to orientate to their immediate environment with or without visual cues (PCA *n* = 30; typical AD *n* = 31; Control *n* = 33). We interpret these findings through a separate group of community‐based PCA participants receiving non‐pharmacological treatment in Osaka (*n* = 10), incorporating low‐ and high‐technology approaches.

**Result:**

When locating target objects, UCL PCA participants were 22% faster at locating targets when surrounding distractors were removed and 14% faster when spacing between target and distractor increased. Typical AD participants showed smaller improvements (6% and 5%). These findings are consistent with reports from Osaka participants, highlighting object localization deficits associated with crowded visual environments. When locating target destinations, UCL PCA and typical AD participants were 12% faster when visual contrast cues were introduced. When orientating to targets, both groups showed exaggerated effects of visual orientation cues compared to controls. Findings are consistent with an increased reliance on particular visual cues and landmarks for orientation, as reported by Osaka participants. Osaka low‐tech management approaches included placing contrasting‐colored stickers on light switches and simplifying the environment. High‐tech approaches include using smartphone GPS when going outdoors, sensor‐equipped keys for automatic door locking/unlocking, and motion‐sensor lights to improve environmental awareness.

**Conclusion:**

Technology‐based evidence indicates PCA patients face significant challenges with object recognition and navigation. A combination of high‐tech and low‐tech approaches may offer substantial benefits, with early interventions being particularly important for effective symptom management.

